# Effect of Oxidative Stress on the Estrogen-NOS-NO-K_Ca_ Channel Pathway in Uteroplacental Dysfunction: Its Implication in Pregnancy Complications

**DOI:** 10.1155/2019/9194269

**Published:** 2019-02-10

**Authors:** Xiang-Qun Hu, Rui Song, Lubo Zhang

**Affiliations:** Lawrence D. Longo, MD Center for Perinatal Biology, Department of Basic Sciences, Loma Linda University School of Medicine, Loma Linda, California 92350, USA

## Abstract

During pregnancy, the adaptive changes in uterine circulation and the formation of the placenta are essential for the growth of the fetus and the well-being of the mother. The steroid hormone estrogen plays a pivotal role in this adaptive process. An insufficient blood supply to the placenta due to uteroplacental dysfunction has been associated with pregnancy complications including preeclampsia and intrauterine fetal growth restriction (IUGR). Oxidative stress is caused by an imbalance between free radical formation and antioxidant defense. Pregnancy itself presents a mild oxidative stress, which is exaggerated in pregnancy complications. Increasing evidence indicates that oxidative stress plays an important role in the maladaptation of uteroplacental circulation partly by impairing estrogen signaling pathways. This review is aimed at providing both an overview of our current understanding of regulation of the estrogen-NOS-NO-K_Ca_ pathway by reactive oxygen species (ROS) in uteroplacental tissues and a link between oxidative stress and uteroplacental dysfunction in pregnancy complications. A better understanding of the mechanisms will facilitate the development of novel and effective therapeutic interventions.

## 1. Introduction

During pregnancy, maternal circulation undergoes significant physiological changes to meet the increased metabolic demand of the growing fetus and the well-being of the mother [1]. Throughout pregnancy, cardiac output rises by increasing heart rate and stroke volume, reaching ~50% above prepregnancy baseline in the third trimester. Systemic vascular resistance decreases by ~20% in the second trimester, leading to reduced mean arterial blood pressure. In addition, blood volume increases by 40-50%. Nevertheless, marked changes also occur at the maternal-fetal interface. The placenta formation and structural and physiological remodeling of uterine arteries lead to the establishment of the low-resistance uteroplacental circulation. In human and sheep, uterine blood flow increases from 20 to 50 ml/min in nonpregnant state to ≥1000 ml/min at near-term pregnancy. Elevated steroid hormones such as 17*β*-estradiol (E_2_*β*) and progesterone are believed to play an important role in the cardiovascular adaptation during pregnancy [2–4].

Aberrant uteroplacental adaptation leads to pregnancy complications such as preeclampsia and intrauterine (fetal) growth restriction (IUGR). These complications are associated with diminished uteroplacental blood flow [5, 6]. Both preeclampsia and IUGR are major causes of maternal and/or fetal morbidity and mortality. Accumulating evidence suggests that preeclampsia and IUGR also have detrimental effects on the health of both the mother beyond pregnancy and offspring. Women with a history of preeclampsia have increased risk of cardiovascular disease [7]. Moreover, offspring born from preeclamptic pregnancy also have high incidence of high blood pressure and stroke later in life [8, 9]. Similarly, IUGR is associated with increased prevalence of metabolic syndrome, diabetes, and cardiovascular disease in later life of offspring [10, 11].

Although the etiologies of preeclampsia and IUGR are not fully elucidated, placental insufficiency (or uteroplacental vascular insufficiency), the inability to deliver an adequate supply of oxygen and nutrients to the fetus due to reduced blood flow to the placenta, is generally considered as a major contributor to the development of these disorders. Soleymanlou et al. revealed a remarkable similarity of global gene expression in hypoxia-treated placenta explants, high-altitude placentas, and preeclamptic placentas [12], implying an important causative role of hypoxia in these complications. This notion is further substantiated by observations in animal models in which gestational hypoxia imitated placental insufficiency, reduced fetal growth, and induced preeclampsia-like symptoms [13–15].

Oxidative stress is defined as an imbalance between oxidants and antioxidants in favor of the oxidants [16]. Prolonged hypoxia is shown to elicit oxidative stress [17]. Consistently, placental insufficiency also promotes oxidative stress in preeclampsia, IUGR, and high-altitude pregnancy [18, 19]. Accumulating evidence suggests a critical role of reactive oxygen species (ROS) in the pathogenesis of pregnancy complications [20, 21]. However, the mechanistic insights into ROS-induced maladaptation of uteroplacental circulation remain largely elusive. In this article, we provide a succinct review of effects of oxidative stress on E_2_*β* signaling pathways in the uteroplacental circulation in pregnancy complications.

## 2. E_2_*β* Signaling and Uteroplacental Circulation in Physiological and Pathophysiological Conditions

### 2.1. Estrogen and Estrogen Receptors (ERs) in Normal Pregnancy and Pregnancy Complications

Both E_2_*β* and its metabolites are essential for the success of pregnancy. Starting from approximately week 9 of gestation, the placenta becomes the primary site of estrogen synthesis involving enzymes such as aromatase (CYP19) and hydroxysteroid 17*β*-dehydrogenases 1 (HSD17B1, 17*β*-HSD1) [22]. Circulating estrogen rises progressively throughout pregnancy, and plasma 17*β*-estradiol (E2*β*) level at term is ~100-fold higher than that in nonpregnant subjects. Similarly, E2*β* metabolites produced by cytochrome P450s and catechol-O-methyltransferase (COMT) such as catecholestradiols also elevated during pregnancy [23]. However, estrogen biosynthesis and metabolism are apparently impaired in pregnancy complications. Maternal plasma E2*β* levels are significantly lower in preeclamptic [24–26] and IUGR [27] pregnancies. Low circulating E2*β* was also observed in high-altitude human and sheep pregnancy [28–30], although one study showed an increase in plasma estrogen [31]. The metabolism of E2*β* is also impaired in preeclampsia, leading to reduced 2-methoxyestrone and 2-methoxyestradiol [25, 32]. It appears that the reduced circulating levels of E2*β* and its metabolites in pregnancy complications are the result of dysregulation of steroidogenic enzyme expression in the placenta. Preeclamptic placenta displayed deficiency of aromatase, HSD17B1, and COMT [24, 25, 32–34]. The impaired estrogen steroidogenesis and metabolism in these disorders are evidently caused by placental insufficiency. Aromatase in cultured human trophoblast cells and in trophoblast cell line JEG-3 was downregulated by hypoxia [24, 35], and the expression of placental aromatase was reduced in a rabbit model of placental ischemia [24]. Aberrant production of E2*β* and its metabolites could contribute to the pathogenesis of pregnancy complications due to their key roles in regulating trophoblast invasion, angiogenesis, and uterine vascular tone, which will be discussed in later sections.

Estrogen produces its plethoric effects *via* interacting with its receptors involving both nongenomic and genomic mechanisms. To elicit genomic actions, estrogen binds to the nuclear estrogen receptor *α* (ER*α*) or estrogen receptor *β* (ER*β*). The receptors become dimerized and bind to the estrogen response element (ERE) located in the target gene promoter, triggering or suppressing gene expression [36]. Estrogen can also activate membrane G-protein-coupled estrogen receptor (GPER, or GPR30) and membrane-associated ER*α* and ER*β*, which in turn stimulate adenylate cyclase to generate cAMP or activate kinases such as tyrosine kinase Src, phosphoinositide 3-kinase (PI3K), extracellular-signal-regulated kinase (ERK), and protein kinase B (PKB or AKT) [37]. Activation of membrane or membrane-associated estrogen receptors can lead both acute and long-term effects. The presence of ER*α*, ER*β*, and GPER in uterine arteries and the placenta has been demonstrated by real-time polymerase chain reaction (PCR), Western blot, and immunohistochemistry [38–41]. The expression of all forms of estrogen receptors in uterine arteries and the placenta increases as pregnancy advances [38–40, 42]. The maintenance or upregulation of ERs in the uteroplacental tissues apparently requires continuous estrogen stimulation. Ovariectomy in sheep reduced ER*β* expression in the endothelium of uterine arteries [42]. In addition, chronic treatment with E2*β in vivo* and ex vivo significantly increased ER*α* expression in uterine arteries [40, 42]. The expression of GPER in HTR8/SVneo cells derived from first trimester extravillous trophoblast and placental extravillous explants was also upregulated by E2*β* [43].

Information on estrogen receptor expression in pregnancy complications is scant, and conflicting observations have been reported. ER*α* expression was described as increased, decreased, or unchanged in the preeclamptic placenta [44–46]. No conclusion could be drawn currently, and more rigorous studies are needed to clarify the discrepancy. The expression of ER*α* in uteroplacental tissues was suppressed in high-altitude pregnancy [40], and hypoxia appeared to be the causative factor responsible for ER*α* downregulation [45, 47]. Defective expression of ER*α* could have profound effects on uteroplacental function including gene expression. Intriguingly, the placental expression of ER*β* appears to be differently affected in preeclampsia and IUGR. Whereas ER*β* expression was reduced in the IUGR placenta [44], an upregulation of ER*β* was observed in preeclamptic placentas [44, 45]. These observations suggest that the etiologies of preeclampsia and IUGR may differ. It remains to be determined whether/how the distinct regulations of ER*β* contribute to the pathogenesis of these two complications. The placental expression of GPER was reduced in preeclamptic pregnancy [43, 48], which may lead to dysfunction of uteroplacental vessels.

### 2.2. Estrogen and the Regulation of Uteroplacental Circulation

Several lines of evidence have implicated a critical role of estrogen in the adaptation of the uteroplacental circulation. First, the high ratio of E2*β* to progesterone in the follicular phase was associated with increased blood to the uterus [49, 50]. Second, reduced uterine vascular resistance and increased uterine blood flow concurred with progressively rising plasma E2*β* levels during pregnancy [51–53]. Third, acute treatment with exogenous E2*β* markedly increased uterine blood flow and/or reduced uterine vascular resistance in nonpregnant animals [54–56]. Fourth, chronic administration of E2*β* into nonpregnant sheep also significantly increased uterine blood flow and/or reduced uterine vascular resistance [57, 58]. *Ex vivo* treatment of uterine arteries from nonpregnant sheep with E2*β* reduced uterine arterial myogenic tone [59]. The chronic effects of E2*β* simulated pregnancy-induced hemodynamic changes in the uterine circulation. Fifth, the nonselective ER*α*/ER*β* antagonist ICI 182,780 reduced the increase in uterine blood flow induced by exogenous E2*β* in nonpregnant sheep and by endogenous E2*β* in the follicular phase of nonpregnant sheep by ~60% [53]. Intriguingly, the same antagonist also lowered basal uterine blood flow in pregnant sheep by 37% [53]. Importantly, E_2_*β* and its metabolites also play an important role in uteroplacental adaptation. E_2_*β*, 2-hydroxyestradiol, 4-hydroxyestradiol, and 4-methoxyestradiol were implicated in angiogenesis by promoting endothelial cell proliferation [60], whereas 2-methoxyestradiol promoted the differentiation of the cytotrophoblast to an invasive phenotype [61].

### 2.3. NO and Ca^2+^-Activated K^+^(K_Ca_) Channels in Regulating Uteroplacental Function

**Figure 1:**
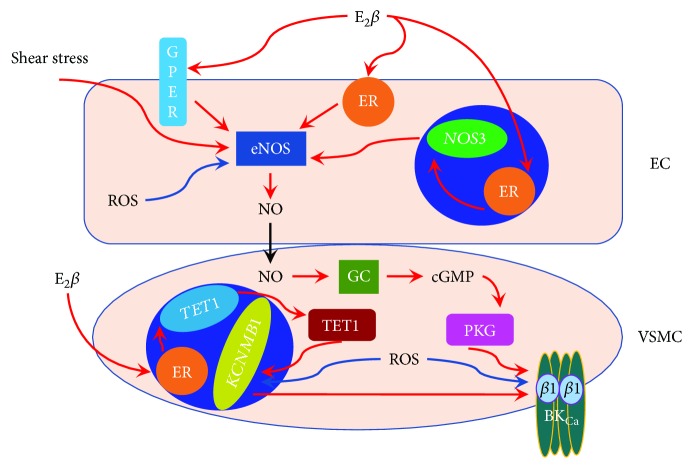
Estrogen (E_2_*β*) regulates uterine artery function partly *via* its actions on endothelial nitric oxide synthase (eNOS) in the endothelial cell (EC) and the large-conductance Ca^2+^-activated K^+^ (BK_Ca_) channel in vascular smooth muscle cell (VSMC) during pregnancy. Shear stress stimulates eNOS activity, leading to increased NO production. E_2_*β* could increase the expression of eNOS in ECs via interacting with nuclear estrogen receptors (ERs) and/or elevate eNOS activity via interacting with the G protein-coupled estrogen receptor (GPER, GPR30) or membrane-associated ER*α* and ER*β*. In addition, E_2_*β* increases the expression of the BK_Ca_ channel *β*1 subunit encoded by *KCNMB1* and channel activity via upregulating ten-eleven translocation methylcytosine dioxygenase 1 (TET1, encoded by *TET1*) in VSMCs. Moreover, the activity of the BK_Ca_ channel can be enhanced by NO-PKG signaling. In pregnancy complications, excessive oxygen species (ROS) impair the estrogen-NOS-NO-BK_Ca_ channel pathway.

Nitric oxide (NO) is a gaseous messenger-generated nitric oxide synthase (NOS). NO contributes to the maintenance of cardiovascular homeostasis by regulating vasocontractility [62]. The large-conductance Ca^2+^-activated K^+^ (BK_Ca_) channel is primarily expressed in vascular smooth muscle cells (VSMCs) and plays a pivotal role in regulating myogenic tone [63]. In VSMCs, the BK_Ca_ channel is a heteromeric assembly of the pore-forming *α* subunit and accessary *β*1 subunits [64]. The *β*1 subunit encoded by *KCNMB1* increases the channel's Ca^2+^/voltage sensitivity. Importantly, the BK_Ca_ channel is one of many targets of NO in the cardiovascular system [64]. Not surprisingly, the NO-cGMP-PKG-BK_Ca_ channel axis is implicated in the adaptation of uteroplacental circulation during pregnancy [65] (Figure 1).

Activation of either of ER*α*, ER*β*, or GPER induced acute vasorelaxation of uterine arteries [66]. The acute estrogen effects in regulating uterine hemodynamics involved stimulation of endothelial NOS (eNOS) activity and increased NO release in endothelial cells (ECs) [38, 67] and activation of BK_Ca_ channels in VSMCs [67]. Stimulation of eNOS activity by estrogen in uterine arterial ECs required phosphorylation of the enzyme at serine 635 and serine 1177 mediated by ER*α* [68]. E2*β* could also directly activate BK_Ca_ channels in uterine arterial VSMCs [67], possibly *via* interacting with the accessory *β*1 subunit [69].

E2*β* could also exert its genomic effect to regulate the expression of both NOS and BK_Ca_ channels in uteroplacental tissues. Expression and function of eNOS [70–72] and the BK_Ca_ channel *β*1 subunit [65, 73, 74] in uterine arteries were increased in the follicular phase and during pregnancy. The upregulation of eNOS and the BK_Ca_ channel *β*1 subunit in uteroplacental circulation during these two physiological states was apparently stimulated by estrogen as chronic treatment with exogenous E2*β* in intact nonpregnant animals [58, 75, 76] and in ex vivo cultured uterine arteries [73] elevated their abundance and activity.

*In vivo* studies revealed distinct contributions of eNOS and the BK_Ca_ channel to basal uterine blood flow in nonpregnant and pregnant sheep. Intrauterine arterial infusion of the NO synthase inhibitor L-nitro-arginine methyl ester (L-NAME) demonstrated minimal contribution of NO to basal uterine blood flow in both nonpregnant and pregnant sheep [77]. However, infusion of the BK_Ca_ channel blocker tetraethylammonium into uterine arteries revealed that at least half of the basal uterine blood flow is maintained by the BK_Ca_ channel in pregnant sheep, whereas the channel did not contribute to basal uterine blood flow in nonpregnant animals [67, 78]. These findings are reinforced by the observations that uterine arterial myogenic tone (i.e., the major constituent of vascular tone) of pregnant subjects was regulated by the BK_Ca_ channel [73], but not by the endothelium [79, 80]. Thus, estrogen-induced eNOS expression and activity during pregnancy are probably responsible for enhanced endothelium-dependent vasorelaxation in uterine arteries in response to given vasodilators [81, 82] and uterine artery remodeling [83], but not for regulating basal uterine vascular tone. In contrast, the upregulation of the BK_Ca_ channel is essential for the reduced uterine vascular tone during pregnancy. In addition, the upregulated BK_Ca_ channel also contributed to blunted vasoconstrictor responses in uterine arteries during pregnancy [65, 84]. Thus, the BK_Ca_ channel in uteroplacental circulation functions as a negative feedback control mechanism to prevent excessive vasoconstriction. Together, these findings reinforced the notion that E2*β*, through its acute and chronic actions on eNOS and BK_Ca_ channels, plays a pivotal role in uteroplacental adaptation.

Expression/activity of placental eNOS in preeclamptic and IUGR pregnancies was reported as either unaltered [85, 86], decreased [44, 87], or increased [88, 89]. Whereas eNOS in placental chorionic plate arteries was downregulated in preeclampsia [90], this enzyme in uterine arteries was upregulated in high-altitude pregnancy [91]. Regardless of uteroplacental eNOS expression status, NO bioavailability in pregnancy complications appeared to be reduced due to substrate deficiency and enzyme inhibition. Both plasma and placental L-arginine levels were reduced in preeclampsia [86, 92]. In addition, the expression of arginase-2, which consumes eNOS's substrate L-arginine, was increased in the placenta and in omental vessels of women with preeclampsia [86, 93]. The increased arginase-2 expression could be imitated by treating human umbilical vein endothelial cells (HUVECs) with preeclamptic plasma [93]. Moreover, HUVECs from IUGR pregnancy also displayed increased arginase-2 expression and activity and placental vessels exhibited impaired eNOS-dependent relaxation [89]. A deficiency of L-arginine would not only reduced eNOS-derived NO but also increase eNOS-mediated superoxide production leading to peroxynitrite (ONOO^−^) formation, evidenced by increased nitrotyrosine staining in villi and maternal vasculature of preeclamptic women [86, 94]. Similarly, nitrotyrosine staining was increased in the syncytiotrophoblast and extravillous trophoblast of high-altitude placenta [95]. Intriguingly, the circulating level of asymmetric dimethylarginine (ADMA), an endogenous NOS inhibitor, also increased in preeclamptic and IUGR pregnancies [96, 97]. Not surprisingly, NOS-dependent relaxation of placental chorionic arteries from IUGR pregnancy was impaired [98]. Moreover, both chronic blockade of NOS with L-NAME or knockout of eNOS in rodents increased maternal blood pressure and reduced fetal growth [99, 100], partly due to impaired uteroplacental vessel remodeling [101].

The expression and function of the BK_Ca_ channel in uteroplacental vessels are also impaired in pregnancy complications. It appears that the *β*1 subunit of the channel is selectively targeted, whereas the *α* subunit remains unaffected in these disorders. The BK_Ca_ channel *β*1 subunit was downregulated in placental chorionic plate arteries in preeclampsia [102] and uterine arteries in high-altitude pregnancy [103]. High-altitude pregnancy also suppressed the ability of estrogen to upregulate the expression of the BK_Ca_ channel *β*1 subunit in uterine arteries [103], leading to increased uterine arterial myogenic tone. BK_Ca_ channel-mediated vasorelaxation was also reduced in both pathological conditions. The impact of high-altitude pregnancy on the BK_Ca_ channel was simulated by ex vivo hypoxia [104], implicating a causative role of hypoxia in the downregulation of the BK_Ca_ channel *β*1 subunit. In a preeclampsia-like murine model induced by autoantibodies against angiotensin II type 1 receptor (AT1-AA), the expression of the BK_Ca_ channel *β*1 subunit and channel activity in mesenteric arteries was also reduced [105].

The intermediate-conductance (IKs) and small-conductance (SKs) Ca^2+^-activated K^+^ channels are predominantly expressed in ECs and also mediate endothelium-dependent vasodilation [106]. The endothelium-derived hyperpolarizing factor (EDHF) causes hyperpolarization of VSMCs by activating IKs and SKs. Both IKs and SKs are expressed in uteroplacental tissues [90, 107, 108]. IKs and SKs are also expressed in VSMCs of uterine and placental chorionic plate arteries in addition to their expression in ECs [90, 107]. In the uteroplacental system, IKs and SKs participated in the regulation of contractility of uterine and placental vessels [90, 107, 109]. Moreover, SK3 was also involved in regulating uterine vascular remodeling and placental vascularization [110, 111]. Like BK_Ca_ channels, E_2_*β* is required to maintain and to upregulate the expression and function of SKs in vasculature. Pregnancy *via* estrogen's action upregulated the expression of SK2 and SK3 in uterine arteries [107]. Ovariectomy reduced SK3 activity in ECs and ablated the channel's role in EDHF-mediated vasorelaxation in nonuterine arteries [112].

The expression and function of IK1, SK2, or SK3 in uteroplacental vessels and umbilical vessels were downregulated in high-altitude pregnancy and preeclampsia [90, 107, 113] as well as in a rat model of preeclampsia induced by testosterone [108]. Given the important role of estrogen in the regulation of IKs and SKs in uteroplacental circulation, it is anticipated that impaired E_2_*β*-ER signaling could contribute to the downregulation of these ion channels in high-altitude and preeclamptic pregnancies.

Together, evidence presented in this section demonstrated critical roles of both estrogen synthesis and metabolism in the adaptation of uteroplacental circulation. Preeminently, E_2_*β* and its metabolites contribute to this adaptive process by promoting angiogenesis, trophoblast invasion, and remodeling and by lowering uterine vascular tone through upregulating activity and/or expression of both eNOS and K_Ca_ channels. However, the E_2_*β*-NOS-NO-K_Ca_ channel pathway is disrupted in pregnancy complications, which could contribute to the pathogenesis of these disorders.

## 3. Oxidative Stress and Pregnancy Complications

### 3.1. Cellular Sources of ROS and Antioxidant Defense

ROS are oxidants formed during oxygen metabolism, primarily produced during oxidative phosphorylation in the mitochondria and by oxidases such as NADPH oxidases (NOXs) and xanthine oxidase (XO) as well as uncoupled NOS [114, 115]. ROS include free radicals such as superoxide (O_2_^**·**-^) and hydroxyl radical (^**·**^OH) and nonradical hydrogen peroxide (H_2_O_2_). In order to maintain redox hemostasis, mammalian cells have developed enzymatic and nonenzymatic defense mechanisms to balance the oxidative state. The major antioxidant enzymes involved in detoxifying ROS include superoxide dismutase (SOD), catalase, glutathione peroxidase (GPx), and peroxiredoxin (Prx) [116]. Nonenzymatic antioxidants include metabolic products such as glutathione (GSH), uric acid, and melatonin [117, 118].

ROS at low levels can act as intracellular second messengers to modulate cellular responses. The very short lifetime and diffusion distance of O_2_^**·**-^ and ^**·**^OH make them unsuitable to function as signaling molecules. In contrast, H_2_O_2_ mediates reversible oxidation of cysteine residues in proteins, which can alter protein activities and functions [119]. These proteins include enzymes (i.e., mitogen-activated protein kinases (MAPKs), tyrosine kinases, and protein tyrosine phosphatases) and transcription factors (i.e., activator protein-1 (AP-1), nuclear factor kappa-light-chain-enhancer of activated B cells (NF-*κ*B), and hypoxia-inducible factor 1 (HIF-1)). When ROS production overwhelms the intrinsic antioxidant defense, due to either increased ROS formation or reduced ability to neutralize ROS or both, oxidative stress arises. As a consequence, ROS attack cellular components, leading to potential cell/tissue damage.

### 3.2. Normal Pregnancy Is a Mild State of Oxidative Stress

The metabolic activity of the placenta is high in order to meet the growth of both the placenta and the fetus, leading to increased ROS production during normal pregnancy. It has long been proposed that pregnancy is a state of oxidative stress [120, 121]. This notion is supported by the following observations: (1) increased levels of superoxide O_2_^**·**-^, 8-iso-prostaglandin F_2_*α* (8-iso-PGF_2_*α*), and malondialdehyde (MDA) in the circulation and the placenta [122–124]; (2) reduced circulating expression and activity of enzymatic antioxidants such as SOD, GPx, and catalase [123, 125]; and (3) decreased levels of nonenzymatic antioxidants including uric acid, vitamin C, vitamin E, and GSH [123, 126]. Notably, the increased ROS production in early pregnancy plays an important role in trophoblast proliferation, differentiation, invasion, and angiogenesis [127, 128]. As gestation advances, placental SODs and catalase as well as total antioxidant capacity also increase [129, 130], which counters the increased ROS generation. Thus, a relatively physiological balance between oxidants and antioxidants is maintained in normal pregnancy.

### 3.3. Pregnancy Complications Are Associated with Heightened Oxidative Stress

Both acute and chronic hypoxia has been shown to elevate ROS [17, 131]. Mitochondria and NOXs are the major sources of ROS in response to oxygen deprivation [132–134]. Placental insufficiency is believed to be a critical element in the pathogenesis of preeclampsia and IUGR. Not surprisingly, these disorders display heightened oxidative stress compared to normal pregnancy. Apparently, both overproduction of ROS and reduction of antioxidant defense contribute to the heightened oxidative stress in pregnancy complications. The activity and expression of oxidant enzymes such as NOXs and XO increased in the preeclamptic placenta and/or circulation [135–137]. In contrast, levels and activity of circulating and placental antioxidant enzymes such as SOD, catalase, and GPx as well as thioredoxin (Trx) were decreased in preeclamptic and IUGR pregnancies [135, 138, 139]. Similarly, activities of SOD, GPx, and Trx reductase (TrxR) were reduced in placentas from high-altitude pregnancy [95]. Moreover, mitochondria in the placenta became dysfunctional in pregnancy complication. Mitochondria appear to be damaged as evidenced by swelling and broken cristae in the preeclamptic placenta [140]. Respiratory chain enzyme expression and activity of mitochondrial complexes were suppressed in preeclamptic and IUGR placentas as well as in placentas from high-altitude pregnancy [140–142], uncoupling respiration from oxidative phosphorylation. Furthermore, circulating and placental nonenzymatic antioxidants including GSH, vitamin C, and melatonin were lower in preeclampsia and IUGR [139, 143, 144]. Concomitantly, both complications exhibited higher ROS [124, 145, 146] and oxidative stress markers [147–149] in the circulation and the placenta, leading to lipid peroxidation and oxidative DNA damage [144, 149]. Increased nitrotyrosine immunostaining was observed in villous vessels of the placenta [94, 135] and systemic vessels [150] in preeclamptic pregnancy, suggesting that preeclampsia promotes NOS uncoupling and OONO^−^ generation.

### 3.4. Animal Models Replicate Oxidative Stress in Pregnancy Complications

The elevated oxidative stress in preeclampsia and IUGR has been imitated in animal models. Increased placental O_2_^**·**-^ was observed in an eNOS^−/−^ mouse model of fetal growth restriction [151]. The reduced uterine perfusion pressure model reduced levels of SODs and GPx, increased levels of MDA, and decreased mitochondrial complexes I and II expression [152–154] in rat placentas. Rodent models of preeclampsia and/or IUGR also promoted eNOS uncoupling in the aorta and placenta [14, 155] and decreased placental GSH content [156]. Therefore, similar to human pregnancy complications, an imbalance between oxidant and antioxidant systems apparently accounts for the heightened oxidative stress in these animal models and hypoxia appeared to be a major cause of the heightened oxidative stress in these models. Increased uterine arterial ROS generation was detected in a sheep model of high-altitude pregnancy due to increased NOX2 expression, which could be replicated by ex vivo hypoxic treatment of uterine arteries [157]. Naïve high-altitude pregnant sheep exhibited higher circulating MDA than low-altitude pregnant sheep and native high-altitude pregnant sheep [30]. In addition, gestational hypoxia increased levels of 4-hydroxynonenal (4-HNE), a lipid peroxidation product, in rat placentas [158].

In conclusion, oxidative stress is an inherent feature of normal pregnancy and plays an important role in the development of the placenta. The uteroplacental system is particularly vulnerable to oxidative stress. When unchecked, oxidative stress becomes augmented and could give rise to pathological conditions such as preeclampsia and IUGA, harming both the mother and the fetus. Therefore, oxidative can play both physiological and pathological roles in the progression and outcome of pregnancy.

## 4. Regulation of E_2_*β* Production and E_2_*β* Signaling Pathway by ROS in Pregnancy Complications

As aforementioned, E_2_*β* is an essential element in the adaptation of the uteroplacental circulation during pregnancy. Given heightened oxidative stress in preeclampsia, IUGR and high-altitude pregnancy, and diverse effects of ROS on macromolecules, it is not surprising that excessive ROS plays a critical role in the pathogenesis of these complications by disrupting the E_2_*β* signaling pathway. ROS could directly or indirectly exert their detrimental effects on the targets, and their actions could be acute or chronic. Unfortunately, there is limited information regarding the impacts of ROS on the E_2_*β* signaling pathway in uteroplacental circulation under pathophysiological conditions. In this section, findings from both uteroplacental and nonuteroplacental tissues/cells will be discussed.

### 4.1. ROS and Estrogen Synthesis

Aromatase and HSD17B1, two key enzymes in estrogen biosynthesis catalyze the interconversion between testosterone and E2*β* and between estrone and E2*β*, respectively, using cofactors NADPH [159, 160]. In fact, NADPH is a key component against cellular oxidation. Maintaining an adequate NADPH/NADP^+^ ratio is essential to activities of these enzymes and E2*β* generation. In HUVECs, high glucose elevated ROS [161] but reduced the NADPH level [162]. Lowering the NADPH/NADP^+^ ratio markedly reduced the conversion of estrone to E2*β* in HEK293 cells [163]. Interestingly, the reduced E2*β* level in preeclamptic placental explants was mimicked by the treatment of placental explants from normal pregnancy with H_2_O_2_ [164]. Moreover, H_2_O_2_ treatment of the homogenate of the human ovary suppressed aromatase activity, which could be prevented by GPx [165]. These observations suggest that oxidative stress could impair estrogen synthesis by suppressing key enzyme activities.

### 4.2. ROS and Estrogen Receptor Expression

ER*α* expression is also subject to ROS modulation. In general, the expression of ER*α* is negatively regulated by ROS. The following observations were made in cancer cell lines. In MCF-7 cells, a brief treatment with glucose oxidase, which catalyzes the oxidation of glucose to H_2_O_2_ and D-glucono-*δ*-lactone, resulted in marked ER*α* level reduction 24 hours after the treatment [166]. Chronic (16 hours) H_2_O_2_ treatment of ZR-75-1 cells also decreased ER*α* protein level [167]. The detrimental effect of H_2_O_2_ on ER*α* expression could be normalized by increasing antioxidant capacity. Overexpression of Prx-1, a H_2_O_2_ scavenger, ablated H_2_O_2_-induced downregulation of ER*α*, whereas inhibition of Prx-1/2 activity with adenanthin promoted ER*α* downregulation [167].

### 4.3. ROS and NO Production

NOS are also regulated by ROS. ROS affect NO production apparently through altering eNOS expression/activity and eNOS cofactors. In HUVECs, H_2_O_2_ treatment for 2 hours increased eNOS phosphorylation of serine 1177 and enzyme activity, whereas catalase did the opposite [168]. However, H_2_O_2_ was found to decrease NO bioavailability in porcine aortic ECs by inactivation of eNOS cofactors without altering enzyme activity [169]. Long-term treatment with H_2_O_2_ or superoxide treatment resulted in downregulation of eNOS in HUVECs [170, 171]. NOXs appeared to be major sources of ROS responsible for eNOS downregulation. HUVECs from women with preeclampsia exhibited NOX2 upregulation and eNOS downregulation [113]. In addition, the upregulation of NOX4 by angiotensin II and high glucose promoted eNOS uncoupling, leading to increased generation of O_2_^**·**-^ and OONO^−^ in glomerular mesangial cells [172, 173]. Thus, it is expected that inhibiting oxidant generation or enhancing antioxidant defense could potentially normalize the adverse effect of ROS on eNOS. As expected, eNOS expression was partially rescued or restored by NOX inhibitor apocynin or overexpression of SOD2 [113, 174]. Administration of the GSH synthase inhibitor buthionine sulfoximine into rats decreased total GSH level in the liver, reduced urinary excretion of NOx, and increased nitrotyrosine staining in the kidney without altering renal eNOS level [175].

### 4.4. ROS and K_Ca_ Channels

ROS display complex actions toward the BK_Ca_ channel. H_2_O_2_ could be stimulatory or inhibitory on BK_Ca_ channel activity depending on experimental conditions. H_2_O_2_ increased BK_Ca_ channel activity in human and porcine artery VSMCs and HUVECs [176–178], whereas it decreased BK_Ca_ channel-mediated currents in porcine renal artery ECs and vascular smooth muscle-type BK_Ca_ channel reconstituted in HEK293 cells [179]. A study by Tang et al. revealed that both cysteine and methionine residues of the BK_Ca_ channel were subject to redox modulation [180]. Interestingly, oxidation of cysteine and methionine produced opposite regulations of BK_Ca_ channel activity. Whereas cysteine oxidation decreased BK_Ca_ channel currents, methionine oxidation increased channel activity. Moreover, oxidation of a cysteine residue near the Ca^2+^ bowl of the BK_Ca_ channel *α* subunit by H_2_O_2_ almost abolished physiological activation of the channel [181]. It is likely that distinct actions of H_2_O_2_ on the BK_Ca_ channel resulted from selectively targeting cysteine and methionine residues. Whereas O_2_^.-^ did not alter currents mediated by the BK_Ca_ channel, ONOO^−^ exhibited an inhibitory effect on BK_Ca_ channel activity [182, 183]. It appears that the BK_Ca_ channel in uterine artery VSMCs of high-altitude pregnant sheep is under tonic inhibition by ROS. An acute application of antioxidants such as N-acetylcysteine (NAC), the NOX inhibitor apocynin, and the synthetic SOD/catalase mimetic EUK-134 partially reversed gestational hypoxia-induced suppression of BK_Ca_ channel-mediated currents and vasorelaxation [104, 184]. As NOX2 was upregulated in gestational hypoxia, the superoxide generated by this enzyme and its dismutation product H_2_O_2_ probably contributed to the gestational hypoxia-induced suppression of BK_Ca_ channel activity/function in uterine arteries [157]. IK channel-mediated currents in HUVECs were also inhibited by the superoxide donors, xanthine/xanthine oxidase (X/XO) mixture [185].

In addition to direct modulation of K_Ca_ channel activity, ROS also exert a significant impact on the expression of K_Ca_ channels. High-altitude pregnancy increased uterine vascular tone owing to NOX2 overexpression and *KCNMB1* downregulation as well as decreased BK_Ca_ channel activity [103, 157]. These detrimental effects could be simulated by ex vivo hypoxic treatment of uterine arteries of low-altitude pregnancy [104]. A cause-and-effect relationship was established by the observation that antioxidants apocynin and NAC largely eliminated gestational hypoxia-induced reduction of *KCNMB1* expression and channel activity [104, 157]. In addition, estrogen-induced upregulation of the BK_Ca_ channel *β*1 subunit and channel activity in uterine arteries was eradicated by gestational hypoxia, which was restored by NAC in ex vivo experiments [104, 184]. Similarly, preeclampsia reduced the expression of *KCNMB1* along with upregulation of NOX2 and superoxide in HUVECs [113]. Importantly, the *KCNMB1* downregulation was partially rescued by treating cultured HUVECs with apocynin [113]. The *KCNMB1* downregulation appeared to be directly induced by ROS. Exposure of the cultured human coronary artery VSMCs to H_2_O_2_ for 12 hours led to reduced *KCNMB1* expression [186]. These observations signal a contributing role of ROS in the dysfunction of the BK_Ca_ channel in uteroplacental circulation. Targeting *KCNMB1* expression by ROS is also observed in diabetes. The BK_Ca_ channel *β*1 subunit protein level was downregulated in diabetic mouse aorta, which was accompanied by increased expression of NOX1 and NOX4, decreased expression of SOD and catalase, and elevated O_2_^·-^ generation [186].

The expression of SK and IK channels is also regulated by ROS in pregnancy complications. Pregnancy/estrogen-induced upregulation of SK2 (K_Ca_2.2) and SK3 (K_Ca_2.3) channel expression/activity in ovine uterine arteries was diminished at high altitude [107], and a causative role of ROS was evidenced by the reversal of gestational hypoxia-induced detrimental effects with NAC [184]. Treatment of human uterine microvascular ECs with serum from preeclamptic women also reduced SK3 and IK1 expression, which was reversed by silencing NOX4 with siRNA or treatment with a membrane-permeable SOD [187]. The reduced expression of SK3 and IK1 (K_Ca_3.1) in the placenta, umbilical vessels, and HUVECs was also associated with the upregulation of NOX2 or NOX4 and heightened oxidative stress in preeclamptic pregnancy [113, 187, 188]. The contributing role of ROS to the downregulation of SK_Ca_ and IK_Ca_ channels was substantiated based on the following findings: (1) restoration of channel expression by antioxidants such as apocynin, tempol, and tiron and (2) simulation of the downregulation by oxidants such as superoxide generated by exogenous X/XO mixture and H_2_O_2_ [113, 188, 189].

Overwhelming evidence suggests that the E_2_*β*-NOS-NO-K_Ca_ channel pathway in uteroplacental tissue is a target of oxidative stress in pregnancy complications. Overall, excessive ROS inhibited E_2_*β* synthesis and estrogen receptor expression. In addition, NOS and K_Ca_ channel expression/activity could also be suppressed by oxidative stress, leading to reduced NO bioavailability and impaired K_Ca_ functions.

## 5. The Interplay among Hypoxia, ROS, and Epigenetic Modifications in Pregnancy Complications

Although it is now well-recognized that placental insufficiency and oxidative stress are important contributors to the pathogenesis of preeclampsia and IUGR, the mechanisms underlying their actions in these complications are not fully resolved. Recent studies have identified epigenetic modifications as important mechanisms underlying various human diseases [190]. In this section, we will try to establish a link among hypoxia, ROS, and epigenome in preeclampsia and IUGR.

### 5.1. ROS in O_2_ Sensing

HIFs are transcription factors and function as master regulators of cellular responses to hypoxia. HIFs are heterodimers composed of a HIF-*α* subunit (HIF-1*α* and HIF-2*α*) and a constitutively expressed HIF-1*β* subunit. Under normoxia, HIF-*α* subunits are hydroxylated on proline residues by the O_2_-dependent prolyl hydroxylases (PHDs), resulting in ubiquitination and successive proteasomal degradation by the von Hippel–Lindau protein (pVHL) E3-ubiquitin ligase. In hypoxia, PHD activity is suppressed. Subsequently, HIF-*α* is accumulated, translocated into the nucleus, and dimerized with HIF-1*β*, leading to gene expression by binding to hypoxia-responsive element (HRE) in the promoter of the target gene. Interestingly, ROS appear to participate in cellular oxygen sensing and hypoxic activation of HIFs. ROS generated by mitochondrial complex III in response to hypoxia were found to stabilize HIF-1*α* [132, 191]. The stabilization of HIF-1*α* was mimicked by exogenous H_2_O_2_ and by genetic suppression of SOD2 under normoxia [191, 192]. However, HIF-1*α* stabilization was attenuated by silencing Rieske iron-sulfur protein of complex III and by enzymatic and nonenzymatic antioxidants [193–195]. ROS produced by NOXs could also led to accumulation of HIF-1*α* [196, 197] and HIF-2*α* [198, 199]. ROS stabilized HIF-*α* apparently through suppressing the ability of the PHDs to hydroxylate HIF-*α* protein [200]. ROS-mediated stabilization of HIFs thus constitutes an important mechanism for hypoxia to stimulate gene expression.

### 5.2. Crosstalk between ROS and Epigenome

Whereas genome confers genetic information for making and maintaining an organism, the epigenome describes all the chemical modifications to DNA and histone proteins. Epigenetic modifications of the genome determine how the information in genes is expressed by switching genes on and off without altering the DNA sequence. The major mechanisms of the epigenetic modification include DNA methylation, histone modifications, and noncoding-RNA-based silencing [201]. Several lines of evidence suggest existence of a crosstalk between ROS and epigenetic modifications. ROS are found to promote DNA hypermethylation by altering DNA methylation/demethylation machineries and enzyme recruitment. In vitro studies demonstrated that H_2_O_2_ treatment increased expression/activity of DNA methyltransferases (DNMTs) [202–204], although many of these studies were conducted in cancer cell lines. In addition, H_2_O_2_ could facilitate DNA methylation by recruiting DNMT1 to the CpG sites in gene promoters [203, 205]. The linking of ROS induced by hypoxia and other stimuli to DNA hypermethylation was further confirmed by findings that antioxidants such as NAC and apocynin were able to prevent both ROS-induced global methylation or specific gene methylation [202, 206, 207] and upregulation of DNMTs [202]. ROS could also impair DNA demethylation. In a cell-free system, H_2_O_2_ suppressed enzymatic activity of ten-eleven translocation (TET) dioxygenase [208]. The catalytic activity of TETs requires vitamin C and Fe^2+^ as cofactors [209, 210]. To maintain an active dioxygenase enzyme, vitamin C is required to reduce Fe^3+^ to Fe^2+^. Thus, vitamin C depletion in pregnancy complications [144, 211] would reduce TET activity. Histone modifications are also subject to ROS regulation. It is found that increasing oxidative stress by H_2_O_2_ upregulated histone deacetylase 1 (HDAC1) in cancer cell lines [204]. Prolonged treatment with H_2_O_2_ also increased global histone methylation marks H3K4me3 and H3K27me3 in human bronchial epithelial cells [208]. It appears that ROS produced from both mitochondria and NOX promotes microRNA-210 (miR-210) generation. Whereas Nox4 siRNA partially decreased hypoxia-induced miR-210 expression, mitochondrial complexes I and III inhibitors rotenone and antimycin increased miR-210 biogenesis in adipose-derived stem cells [212].

Conversely, ROS production could be altered by epigenetic modifications of genes for enzymatic oxidants and antioxidants. It appears that hypermethylation promotes oxidative stress, whereas demethylation boosts antioxidation. In human pulmonary arterial hypertension, a CpG island in an enhancer region of intron 2 and another in the promoter of SOD2 were hypermethylated in pulmonary arterial smooth muscle cells (PASMCs) owing to upregulation of DNMT1 and DNMT3b, leading to downregulation of the antioxidant enzyme [213]. Similarly, hypoxia also reduced SOD2 expression in the rat carotid body via hypermethylation of a single CpG dinucleotide close to the transcription start site [214]. H_2_O_2_ promoted methylation of a CpG island in the catalase promoter and downregulated catalase [215]. TET1 deficiency produced by TET1 siRNA enhanced H_2_O_2_-induced increase apoptosis of cerebellar granule cells [216], suggesting that TET1-mediated demethylation may upregulate antioxidant mechanisms to counter oxidative stress. Histone modification also contributes to the hemostasis of the oxidant-antioxidant system. The expression/activity of SOD3 in the lung from human idiopathic pulmonary arterial hypertension was reduced, and this downregulation could be reversed by the treatment of PASMCs with class I HDAC inhibitors or HDAC3 siRNA [217], suggesting that histone deacetylation negatively regulates SOD3 expression. In contrast, histone deacetylation mediated by HDAC3 upregulated NOX4 in HUVECs as HDAC3 siRNA and pan-HDAC inhibitor scriptaid reduced NOX4 expression [218]. Furthermore, miRs also participate in the regulation of mitochondrial metabolism and function. The downregulation of iron-sulfur cluster assembly enzyme (ISCU) in mitochondria by miR-210 in hypoxia would block electron exit from complex I, promoting its leakage to generation of ROS [219]. Overall, it appears that there exists a positive feed-forward loop between ROS generation and epigenetic modifications.

### 5.3. Epigenetic Mechanisms in Regulating Uteroplacental Circulation during Normal Pregnancy

In sheep, the upregulation of ER*α* in uterine arteries was conferred by an epigenetic mechanism [220]. The specificity protein 1- (Sp1-) binding site (Sp1_−520_) at the promoter of the ER*α* encoding gene *ESR1*, to which Sp1 or Sp1-ER*α* binds, was essential for E2*β*-stimulated promoter activity. The CpG dinucleotide of this site was hypermethylated in nonpregnant animals, and the gene is thus kept quiescent. However, the Sp1 site became less methylated in pregnant animals and enabled the expression of the gene, leading to increased ER*α* mRNA and protein abundance in uterine arteries and subsequent attenuation of uterine vascular tone.

E2*β* also epigenetically upregulates *KCNMB1* expression in uterine arteries [221, 222]. Similar to ER*α*, the CpG dinucleotide in the Sp1-binding site (-380) at the promoter of *KCNMB1* was highly methylated in uterine arteries of nonpregnant sheep, resulting in gene silence. During pregnancy, E2*β* through ER*α* stimulated *TET1* (TET1 encoding gene) promoter activity and gene expression. The upregulation of *TET1* in turn promoted Sp1_−380_ demethylation of the *KCNMB1* promoter. Consequently, the expression of *KCNMB1* and the activity of the BK_Ca_ channel increased in uterine arteries, leading to reduced myogenic tone.

### 5.4. Aberrant Epigenetic Modifications in Pregnancy Complications

Epigenetic mechanisms play an important role in the pathophysiological processes of pregnancy complications. Global hypermethylation was observed in preeclamptic placenta [223, 224]. In addition, various genes including *ESR1* and *KCNMB1* in the uterine arteries of high-altitude pregnant sheep [52, 220, 221, 225] and *IGF1*, *HSD11B2*, *H19*, and *HLA-G* in the placenta from preeclamptic and IUGR pregnancies [224, 226, 227] were hypermethylated. The increased methylation in the uteroplacental tissues was accompanied by upregulation of DNMT1 and DNMT3b expression/activity [224, 225, 227, 228] and downregulation of TET1, TET2, and TET3 expression [52, 227, 229, 230]. Pregnancy complications also alter histone modification in the placenta. JMJD6 histone demethylase activity was suppressed in preeclamptic placenta [231]. Moreover, miR-210 was also upregulated in both uterine arteries and placenta of high-altitude pregnancy [52, 142]. Increased miR-210 level was also observed in preeclamptic and IUGR placenta [140, 230, 232]. These changes undoubtedly would contribute to the aberrant expression of key elements in the E_2_*β*-NOS-NO-K_Ca_ pathway in uteroplacental circulation.

The aforementioned changes in epigenetic modifications of the uteroplacental system in pregnancy complications are apparently caused by hypoxia/ischemia. HIF-1*α* overexpression in uteroplacental tissues is a characterized feature in pregnancy complications and high-altitude pregnancy [157, 233, 234]. Both ex vivo hypoxia treatment of tissues or pharmacologically induced hypoxia in intact animal models induced the expression of DNMTs and miR-210 [142, 225, 235] and repressed both histone demethylase activity [231] and TETs expression/activity [235, 236]. Although not investigated in the uteroplacental tissues, studies conducted in other tissues/cells suggest that hypoxia-induced alterations in epigenetic machineries is HIF-1*α*-dependent. *DNMT1*, *DNMT3b*, and *miR210* all contain hypoxia-responsive element (HRE) in their promoters, and the binding of HIF-1*α* to HRE stimulates the expression of these genes [237]. Hypoxia *via* HIF-1*α* also induced the expression of histone demethylases JHDM1B/KDM2B and JARID1B/KDM5B, which demethylate the activating mark H3K4me2/3, leading to gene repression [238]. The E_2_*β* metabolite 2-methoxyestradiol is an endogenous HIF inhibitor [239]. The reduced 2-methoxyestradiol level in preeclampsia probably contributes to aberrant epigenetic modifications in uteroplacental tissues due to the relief of HIF inhibition.

**Figure 2:**
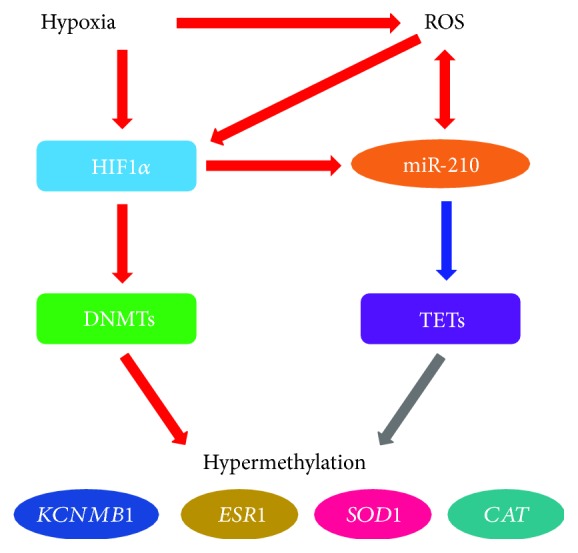
Crosstalk among hypoxia, ROS, and DNA methylation. The cellular responses to hypoxia are primarily mediated by hypoxia-inducible factor-1 (HIF-1). Hypoxia could induce HIF-1 either directly or indirectly through the stabilization of HIF-1*α* by ROS. HIF-1 could upregulate DNA methyltransferases (DNMTs) and miR-210. ROS may be able directly to induce miR-210. TET1 mRNA is a target of miR-210 and is degraded upon the binding of miR-210 to the 3′UTR of the transcript. The upregulation of DNMTs and downregulation of TET1 result in hypermethylation of *ESR*, *KCNMB1*, *SOD1*, and *CAT* (catalase encoding gene) and subsequent gene repression. The repression of *ESR* and *KCNMB1* ultimately increases uterine vascular tone, whereas the repression of *SOD1* and *CAT* elevates ROS. Moreover, miR-210 could also target iron-sulfur cluster scaffold (ISCU) in the mitochondria leading to increased ROS formation. Red arrow: stimulatory effect, blue arrow: inhibitory effect, and grey arrow: indirect action due to TET1 downregulation.

Intriguingly, hypoxia-induced *TET1* repression in uterine arteries was mediated by miR-210 and the binding of miR-210 to the 3′-untranslated region (3′UTR) of TET1 mRNA resulted in degradation of the transcript [52]. The overall effects of upregulation of DNMT3b and downregulation of TET1 in uterine arteries promoted *ESR1* and *KCNMB1* hypermethylation and gene repression [52, 220, 221, 225, 235]. ER*α* and the BK_Ca_ channel are two key elements contributing to reduced uterine vascular tone in pregnancy [59, 73]. Consequently, the downregulation of both ER*α* and the BK_Ca_ channel impaired pregnancy-induced attenuation of uterine vascular tone, leading to maladaptation of uteroplacental circulation [40, 47, 225] (Figure 2). Increased DNA methylation may also contribute to impaired spiral artery remodeling. The downregulation of TET2 reduced *in vitro* trophoblast migration and invasion [230]. The overexpression of miR-210 in the preeclamptic placenta suppressed ISCU and impaired mitochondrial respiration [140, 142, 232]. It is probably that both the miR-210-mediated mitochondrial dysfunction and DNA hypermethylation (indirectly *via* downregulating TETs) disrupt trophoblast invasion and impair spiral artery remodeling in high-altitude pregnancy and pregnancy complications. In addition, miR-210 also targeted potassium channel modulatory factor 1 (KCMF1) and thrombospondin type I domain-containing 7A (THSD7A), which could also contribute to the impaired trophoblast invasion [240, 241]. The expression of *CYP19A1* and *HSD17B1* is also regulated by DNA methylation. Methylation of CpG islands in the promoters of both genes suppressed their expression [242, 243]. Although not examined in the placenta, it is probably DNA methylation-mediated downregulation of aromatase and HSD17B1 also occurs in preeclampsia, IUGR, and high-altitude pregnancy. Furthermore, the expression of *HSD17B1* was downregulated by miR-210 in preeclamptic placenta [33]. The epigenetic modifications of key enzymes in estrogen biosynthesis could then reduce circulating E2*β* level in pregnancy complications.

## 6. Concluding Remarks

Preeclampsia and IUGR are leading causes of maternal and perinatal mortality and morbidity and have great impacts on maternal and offspring health. Unfortunately, there is currently no cure for them. Preeclampsia, IUGR, and high-altitude pregnancy all exhibit uteroplacental hypoxia/ischemia and oxidative stress concurrently. Moreover, these pregnancy complications are associated with altered epigenome. There exist interplays among ROS, HIFs, and epigenome. The ROS-HIF pathway appears to be a potential cause in the changes of epigenetic modifications in these complications. In uterine arteries, HIF-1*α* apparently functions as an important link between ROS and aberrant epigenetic modifications, leading to disrupted E_2_*β*-BK_Ca_ axis and increased uterine vascular tone. In the placenta, the ROS-HIF-epigenome interplay impairs estrogen synthesis, trophoblast invasion, and spiral artery transformation. Both preeclampsia and IUGR are multifactorial disorders. What we know about these complications is only the tip of the iceberg. Further studies are needed to advance our understanding on the pathogenesis of them in order to develop effective therapeutics.

## Abbreviations

3′UTR: 3′-Untranslated region
4-HNE: 4-Hydroxynonenal
8-iso-PGF_2_*α*: 8-Iso-prostaglandin F_2_*α*
ADMA: Asymmetric dimethylarginine
AP-1: Activator protein-1
AT1-AA: Autoantibodies against angiotensin II type 1 receptor
BK_Ca_: Large-conductance Ca^2+^-activated K^+^ channel
cAMP: Cyclic adenosine monophosphate
cGMP: Cyclic guanosine monophosphate
COMT: Catechol-O-methyltransferase
CpG: Cytosine-guanine dinucleotide
CYP19: Aromatase
*CYP19A1*: The gene encoding aromatase
DNMT: DNA methyltransferase
E2*β*: 17*β*-Estradiol
ECs: Endothelial cells
EDHF: Endothelium-derived hyperpolarizing factor
eNOS: Endothelial nitric oxide synthase
ER*α*: Estrogen receptor *α*
ER*β*: Estrogen receptor *β*
ERK: Extracellular signal-regulated kinase
ERE: Estrogen response element
*ESR1*: The gene encoding ER*α*
GPER (GPR30): G-protein-coupled estrogen receptor
GPx: Glutathione peroxidase
GSH: Glutathione
*H19*: The gene encoding imprinted maternally expressed transcript
HRE: Hypoxia-responsive element
HDAC: Histone deacetylase
HIF: Hypoxia-inducible factor
*HLA-G*: The gene encoding major histocompatibility complex, class I, G
H_2_O_2_: Hydrogen peroxide
HRE: Hypoxia-responsive element
*HSD11B2*: The gene encoding hydroxysteroid 11*β*-dehydrogenase 2
HSD17B1 (17*β*-HSD1): Hydroxysteroid 17*β*-dehydrogenases 1
HUVEC: Human umbilical vein endothelial cell
*IGF1*: The gene encoding insulin-like growth factor 1
IK: Intermediate-conductance Ca^2+^-activated K^+^ channel
IUGR: Intrauterine growth restriction
ISCU: Iron-sulfur cluster scaffold
K_Ca_: Ca^2+^-activated K^+^ channel
KCMF1: Potassium channel modulatory factor 1
*KCNMB1*: The gene encoding BK_Ca_ channel *β* subunit 1
L-NAME: L-Nitro-arginine methyl ester or N*^ω^*-nitro-L-arginine methyl ester
MDA: Malondialdehyde
MAPKs: Mitogen-activated protein kinases
miR: MicroRNA
NAC: N-Acetylcysteine
NADP: Nicotinamide adenine dinucleotide phosphate
NADPH: Reduced form of NADP^+^
NF-*κ*B: Nuclear factor kappa-light-chain-enhancer of activated B cells
NO: Nitric oxide
NOS: Nitric oxide synthases
NOX: NADPH oxidase
O_2_: Oxygen
O_2_^**·**-^: Superoxide
^**·**^OH: Hydroxyl radical
ONOO^−^: Peroxynitrite
PASMC: Pulmonary arterial smooth muscle cell
PHD: Prolyl hydroxylases
PCR: Polymerase chain reaction
PI3K: Phosphoinositide 3-kinase
PKB (AKT): Protein kinase B
PKG: Protein kinase G
Prx: Peroxiredoxin
pVHL: von Hippel–Lindau protein
ROS: Reactive oxygen species
siRNA: Small interfering RNA
SK: Small-conductance Ca^2+^-activated K^+^ channel
SOD: Superoxide dismutase
Sp1: Specificity protein 1
TET: Ten-eleven translocation dioxygenase
THSD7A: Thrombospondin type I domain containing 7A
Trx: Thioredoxin
TrxR: Thioredoxin reductase
VSMCs: Vascular smooth muscle cells
X: Xanthine
XO: Xanthine oxidase.
